# Flexible High-Resolution Force and Dimpling Measurement System for Pia and Dura Penetration During *In Vivo* Microelectrode Insertion Into Rat Brain

**DOI:** 10.1109/TBME.2021.3070781

**Published:** 2021-07-16

**Authors:** Lei Chen, Jeremiah P. Hartner, Tianshu Kelly Dong, Annie D.R. Li, Brendon O. Watson, Albert J. Shih

**Affiliations:** Department of Mechanical Engineering, University of Massachusetts Lowell, Lowell, MA 01854 USA and also with the Department of Psychiatry, University of Michigan, Ann Arbor, MI 48105 USA; Department of Psychiatry, University of Michigan.; Department of Mechanical Engineering, University of Michigan.; Department of Mechanical Engineering, University of Michigan.; Department of Psychiatry, University of Michigan.; Department of Mechanical Engineering, University of Michigan.

**Keywords:** Microelectrode, rupture force, dimpling depth, dura and pia penetration, *in vivo* experimental measurement

## Abstract

**Objective:**

Understanding the *in vivo* force and tissue dimpling during micro-electrode implantation into the brain are important for neuro-electrophysiology to minimize damage while enabling accurate placement and stable chronic extracellular electrophysiological recordings. Prior studies were unable to measure the sub-mN forces exerted during *in vivo* insertion of small electrodes. Here, we have investigated the *in vivo* force and dimpling depth profiles during brain surface membrane rupture (including dura) in anesthetized rats.

**Methods:**

A *µ*N-resolution cantilever beam-based measurement system was designed, built, and calibrated and adapted for *in vivo* use. A total of 244 *in vivo* insertion tests were conducted on 8 anesthetized rats with 121 through pia mater and 123 through dura and pia combined.

**Results:**

Both microwire tip sharpening and diameter reduction reduced membrane rupture force (insertion force) and eased brain surface penetration. But dimpling depth and rupture force are not always strongly correlated. Multi-shank silicon probes showed smaller dimpling and rupture force per shank than single shank devices.

**Conclusion:**

A force measurement system with flexible range and *µ*N-level resolution (up to 0.032 *µ*N) was achieved and proved feasible. For both pia-only and dura-pia penetrations in anesthetized rats, the rupture force and membrane dimpling depth at rupture are linearly related to the microwire diameter.

**Significance:**

We have developed a new system with both *µ*N-level resolution and capacity to be used *in vivo* for measurement of force profiles of various neural interfaces into the brain. This allows quantification of brain tissue cutting and provides design guidelines for optimal neural interfaces.

## I. Introduction

The mammalian brain operates via billions of neurons firing action potentials to transmit electrical signals. These neuronal electrical signals are believed to underlie cognitive processes (e.g., perception, motion, memory, sleep) [1], [2]. The action potential signal emitted from single neurons occurs at the milliseconds time scale, with extracellular amplitudes typically smaller than 500 *μ*V in magnitude, and is distinguishable within a 50–150 *μ*m radius of the cell body [3]. To detect and record such small and fast changing live-animal signals so close to single neurons, invasive microelectrode implantation methods have been demonstrated to be effective.

The viability of microwire electrodes for neuronal recordings in live mammals was first demonstrated in 1958 by Strumwasser [4] using 80 *μ*m diameter stainless steel wire electrodes. Since then, a wide variety of materials, including stainless steel (SS) [5], tungsten (W) [6]–[11], Ni-Cr [12], Pt-Ir [13], [14], carbon fiber [15], [16], and conducting polymer [17], have been used. At the same time, development of micro-electro-mechanical systems (MEMS) enabled the use of silicon probes for very high channel count recordings from densely-spaced leads [18]–[21]. Despite that, microwire electrodes remain an important tool for neural recordings due to their lower cost and potentially better unit yield per probe than silicon probes [22] as well as modularity, flexibility, and broad availability. Compared to silicon probes, microwire-based microelectrodes have demonstrated the ability to record activity for months and even years due to the smaller diameter and greater flexibility, which lessens brain tissue inflammation [23].

From the perspective of brain tissue damage and stable chronic recording, a smaller microwire diameter is preferable, but buckling and placement accuracy are major challenges for such thin microwires. Buckling occurs when the force during implantation is larger than the microwire critical buckling load *P* [24], which is calculated by:

(1) $$P=\frac{m\pi^{2}EI}{{L_{U}}^{2}}$$

where *E* is elastic modulus (determined by microwire material), *I* is the second moment of inertia of the microwire (determined by microelectrode geometry), *L*_*U*_ is unsupported length during insertion (could be minimized through extra support and insertion guides [25]), and *m* is the end condition (dependent on buckling mode under specific insertion situation). For example, the carbon fiber microwire has a very small diameter (7 *μ*m), but will buckle if inserted more than 0.75 mm deep into the rat brain tissue without additional support structures due to its small critical buckling load [26]. On the other hand, microwires of larger diameter have higher critical buckling load and will sustain higher insertion force and enable deeper insertion but will cause more damage. This tradeoff means there is a need to minimize wire diameter while still preventing buckling. However, the correlation between microwire diameter/tip shape and insertion force is not well understood, leading to microwires with diameters larger than necessary being used to reach target regions, causing extra brain tissue damage.

Research has been conducted to measure the microwire force profile during brain implantation, though with the limitation of either using mN-rather than *μ*N-resolution devices, thereby limiting our knowledge of the dynamics of in vivo insertion, or measuring outside the context of live brains [27]–[31]. Jensen *et al.* [27] first developed a spiral spring based force sensor and characterized the insertion force of a 50 *μ*m tungsten microwire with sharp tip into in-vitro excised rabbit peripheral nerve. A dimpling force of about 2 mN was reported. The same group measured the insertion force of 50 and 150 *μ*m diameter sharp tungsten microwires into in vivo cerebral cortex of rats [28]. Penetration forces at mN-scale were reported but the commercial strain gauge load cell used had only 1.5 mN accuracy. Haj Hosseini *et al.* [29] utilized a piezoresistive force sensor (0.5 mN accuracy) to measure the insertion force of tungsten microwires (135 *μ*m diameter) and silicon/glass/polyimide tubes at tens of mN level. In vivo insertion force of 100 × 120 *μ*m^2^ silicon probes was measured on an anesthetized Long Evans rat and an awake Macaque monkey and other insertion tests were conducted on explanted brain from freshly slaughtered cow and lamb. Sharp *et al.* [30] evaluated the insertion of blunt and sharp 100 and 200 *μ*m diameter stainless steel microwires into live mouse brain with a high resolution load cell and reported the peak force of 300 to 800 *μ*N, depending on the microwire insertion rate (speed). No data was presented on microwires smaller than 100 *μ*m diameter. More recently, Obaid *et al.* [31] modified a nanoindentation force measurement system for ex-vivo mouse brain penetration through the pia mater as a function of microwire diameter (7.5 *μ*m to 100 *μ*m) and tip geometry (flat, angled, and electro-sharpened). The trend of pia rupture force (insertion force needed to rupture the pia layer) and tissue dimpling depth were linear with the microwire diameter instead of cross-sectional area. No statistical difference was observed between angled and flat tipped microelectrodes. This system provided the highest resolution (nN level) force measurement but was also expensive and required extra sensor control protocol due to the 50 *μ*m working distance limit of the three-plate capacitor force transducer.

To our knowledge, this research field currently lacks force and dimpling data during: 1) in vivo insertion into live brain with miniatured microwire diameters – possibly in part because of the difficulty in measuring such small forces in vivo and 2) in vivo dura mater penetration – likely due to challenges of large force measurement range (tens of mN) needed for dura while keeping high measurement resolution. Factors differentiating in vivo from ex vivo conditions are vibration caused by animal breathing under anesthesia, cardiac pulsations, and probably most importantly, tissue stiffness changes occurring after death. Therefore, without in vivo insertion data, it is difficult to investigate the effect of design parameters (microwire diameter, tip geometry, etc.) under a live-animal environment to determine the optimal microelectrode for minimal damage insertion in animal surgery. Furthermore, with quantitative understanding of the dura penetration process, optimizing design parameters and insertion strategy could potentially enable cellular-scale microelectrode insertion into the brain without dura mater removal [25], thereby removing time-consuming and often dangerous surgical steps and maintaining protection provided by the dura from infection.

In this study, a new force and dimpling measurement system is presented to evaluate the in vivo forces of thin microwires under 100 *μ*m diameter with *μ*N resolution in an anesthetized animal. The system is based on a simple cantilever beam setup made with easily accessible components to achieve high resolution (*<*1 *μ*N) and sampling rate (500 Hz), making it broadly duplicable by the neuroscience community. By using cantilever beams of various sizes, this flexible and reconfigurable system can evaluate both pia-only and dura-pia penetration processes, even with multi-shank silicon-based probes as the insertion device. Taking advantage of the developed system, live-rat force profiles and dimpling depths of microwires with various diameters, materials, and tip shapes were studied.

## II. Methods

### A. Force Measurement System Design

**Fig. 1.**
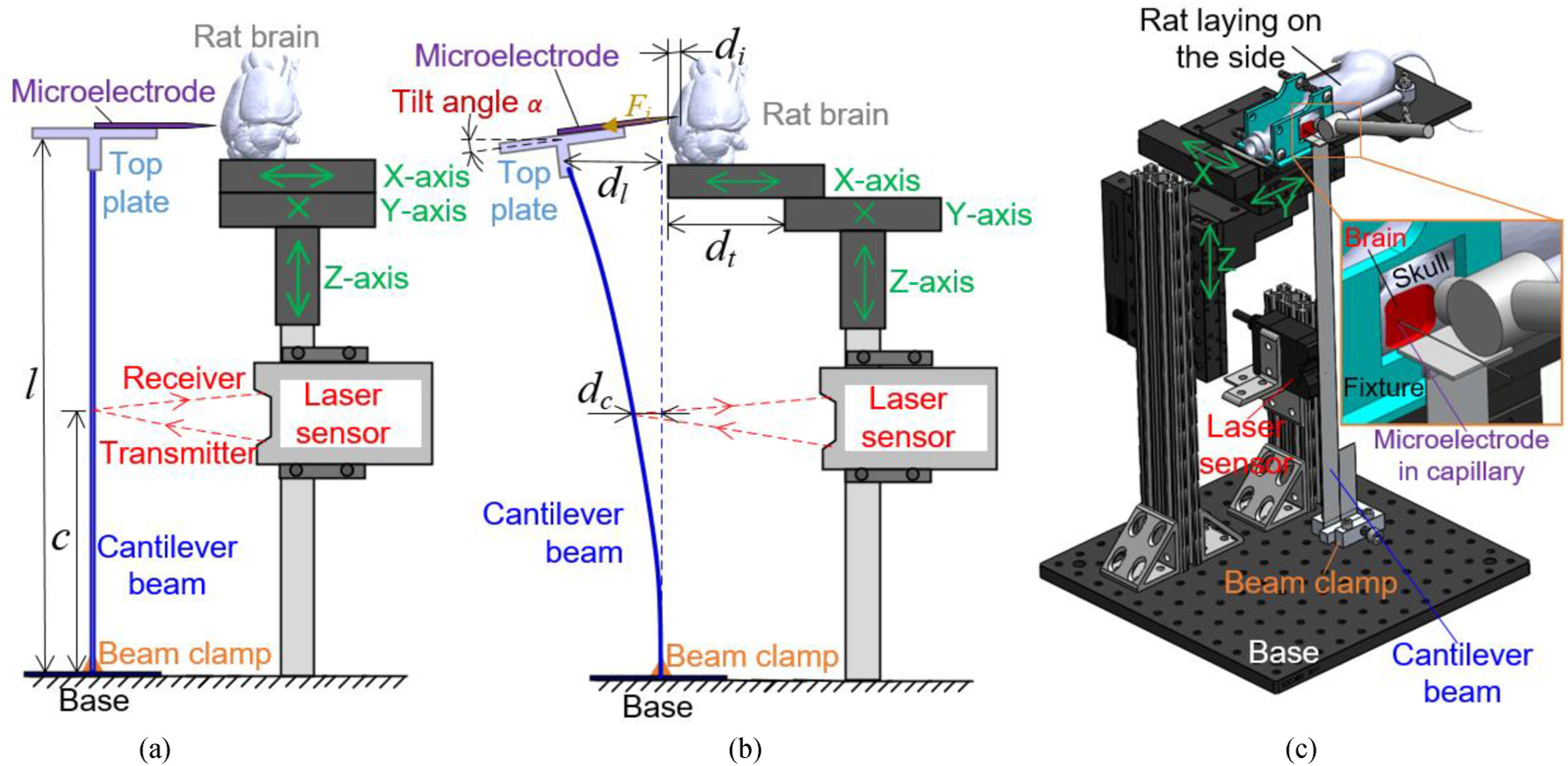
The in vivo microwire force measurement system: (a) conceptual overview, (b) side-view of cantilever beam bending during insertion, and (c) perspective view of experimental setup with anesthetized rat.

A conceptual overview of the force measurement system is shown in Fig. 1(a). The system consisted of a cantilever beam, a 3D-printed top plate on the beam for microwire fixation, and three computer-controlled linear stages (Model 100cri by Siskiyou, Grants Pass, OR) to move the rat and brain along X-, Y-, and Z-axes. A laser displacement sensor was used to measure the cantilever beam deflection during the in vivo microelectrode insertion, as shown in Fig. 1(b). The brain of a live anesthetized rat was exposed and fixed on X-axis linear stage by laying the animal on its side with its head fixed by a 3D-printed fixture (Fig. 1(c)). The vertical cantilever beam setup with the animal on its side was chosen over the more standard top-down insertion orientation to mitigate the effect of gravity on bending of the long and highly flexible and sensitive cantilever beam (to be demonstrated in Section II.B). Stages in Y- and Z-axes positioned the exposed brain region of anesthetized animal to the microwire for in vivo insertion. The X-axis linear stage moved the brain against the microwire by a distance *d*_*t*_. Due to the force (*F*_*i*_) that the microwire encountered during the implantation, the cantilever beam deflected by *d*_*l*_ at the microwire height (*l* from the base of the cantilever beam) and *d*_*c*_ at the laser sensor height (*c* from the base of the cantilever beam). The laser sensor used in this study (Model LK-G10 by Keyence, Osaka, Japan) had a 0.3 mW 650 nm red semiconductor laser and used optical triangulation to measure distance. The sensor had a 0.01 *μ*m measurement repeatability and 2 mm range. The distance *c* was 171 mm. To enable force measurement at different ranges (sub-mN to tens of mN) for various microelectrodes and membrane penetration conditions, four different cantilever beams (Beams #1–4) with parameters listed in Table I were used. These four beams have different widths and thicknesses to achieve various flexural stiffness. The beam flexural rigidity (*EI*) is calculated as:

(2) $$EI=E\frac{bh^{3}}{12}$$

where *E* is the elastic modulus of the beam material (73.1 GPa for Al-2024 and 68.9 GPa for Al-6061), *I* is the moment of inertia of the beam, and *b* and *h* are width and thickness of the beam, correspondingly.

The displacement between the microwire tip and the unde-formed brain surface, *d*_*i*_, which indicates the tissue dimpling before membrane penetration and the insertion depth after tissue rebounding, is:

(3) $$d_{i}=d_{t}-d_{l}$$

where *d*_*l*_ is estimated from the laser sensor measurement of the beam deflection *d*_*c*_. The correlations between *F*_*i*_, *d*_*l*_, and *d*_*c*_ were obtained through cantilever beam calibration (to be elaborated in Section 2.B).

A perspective view of the cantilever beam force measurement system (with Beam #1 installed) and a window of exposed brain for microwire insertion in live anesthetized rat are shown in Fig. 1(c). The microelectrode was glued inside a capillary tube (0.3 mm inner diameter and 0.1 mm wall thickness) with the tip overhang outside. The tube was fixed onto the top just before testing with both ends extended beyond the plate by 10 mm to ensure the weight balance.

### B. Force Measurement System Calibration

This newly developed force measurement system was calibrated through both experiments and finite element modeling (FEM) to correlate *d*_*c*_ with *F*_*i*_ and *d*_*l*_.

**Fig. 2.**
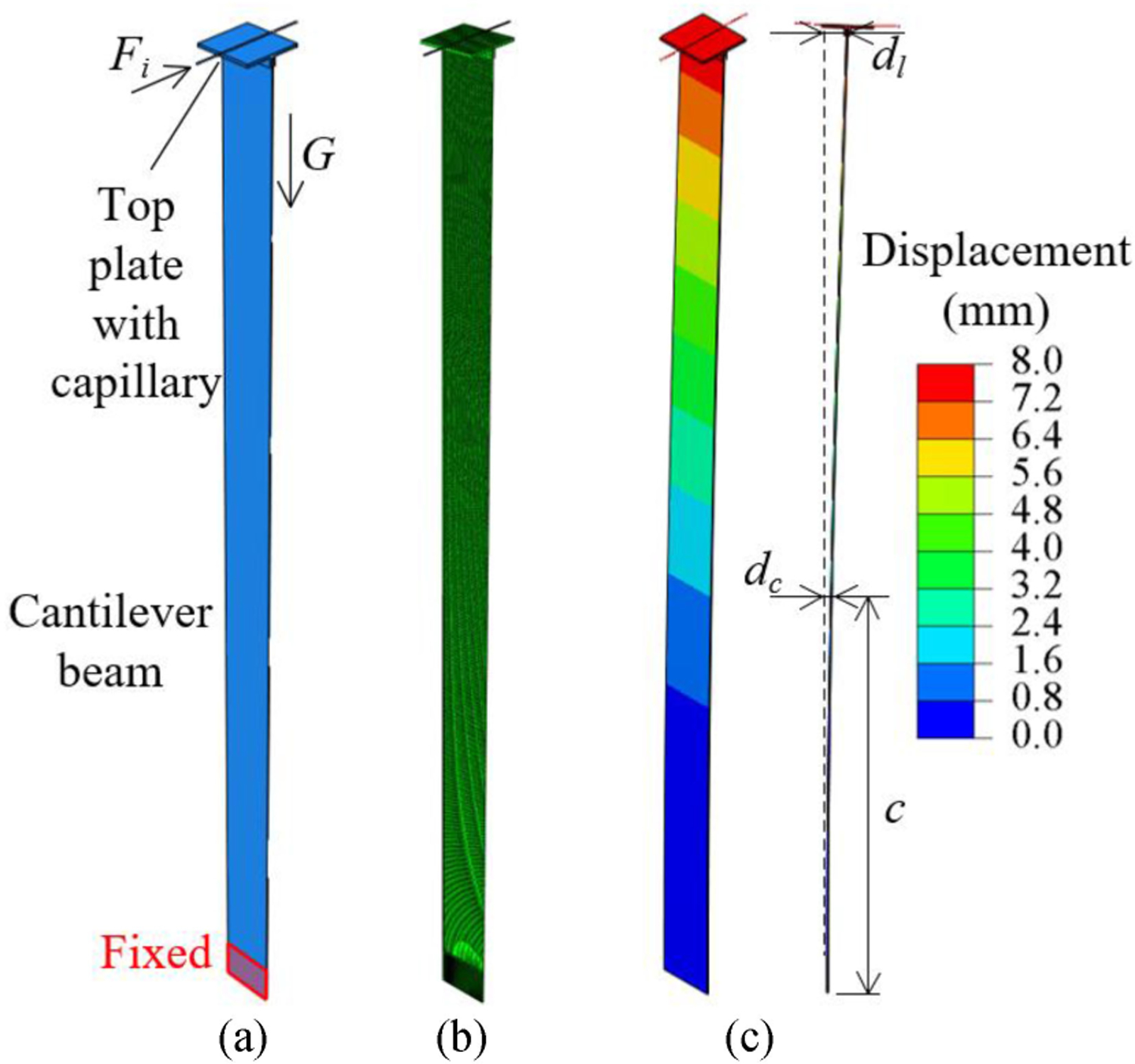
FEM of the cantilever beam system with Beam #1 as example: (a) model configuration overview, (b) FEM mesh setup, and (c) perspective and side views of the beam deflection result under 6 mN load.

A FEM was developed for each one of the four cantilever beams with corresponding top plates and capillary tubes. The effect of the microelectrode weight (less than 0.1 mN) was assumed to be negligible. As shown in the model overview for Beam #1 in Fig. 2(a), bottom of the beam (15 mm high, same as the beam clamp in experiment shown in Fig. 1) was fixed in all directions as boundary condition. The interfaces between the beam, top plate, and capillary tube were tied together in the model to simulate the rigid connection in experiments. Material properties of Al-2024 or Al-6061, borosilicate glass, and 3D-printed parts (using Formlabs^™^ clear resin) were applied to the beam, capillary tube, and 3D-printed top plate correspondingly. The force was applied on the end surface of the capillary tube and gravity of all three components were added. Note that a wider design of Beam #3 led to a wider top plate with larger weight (1.19 g) than the ones used for Beams # 1, 2, and 4 (0.60 g). The magnitude of the force applied was up to 60 mN for Beams #1, #2, and #3 and up to 200 mN for Beam #4. Fig. 2(b) shows the mesh setup of the Beam #1 model with 202155 elements in total. Linear hexahedron elements were assigned to all three parts with average element size of 0.3, 0.8, and 0.1 mm for the beam, top plate, and capillary tube, respectively. Static analysis with steps of incrementally increasing force was conducted for all four beam models using Abaqus v6.11–1 FEM software.

**Fig. 3.**
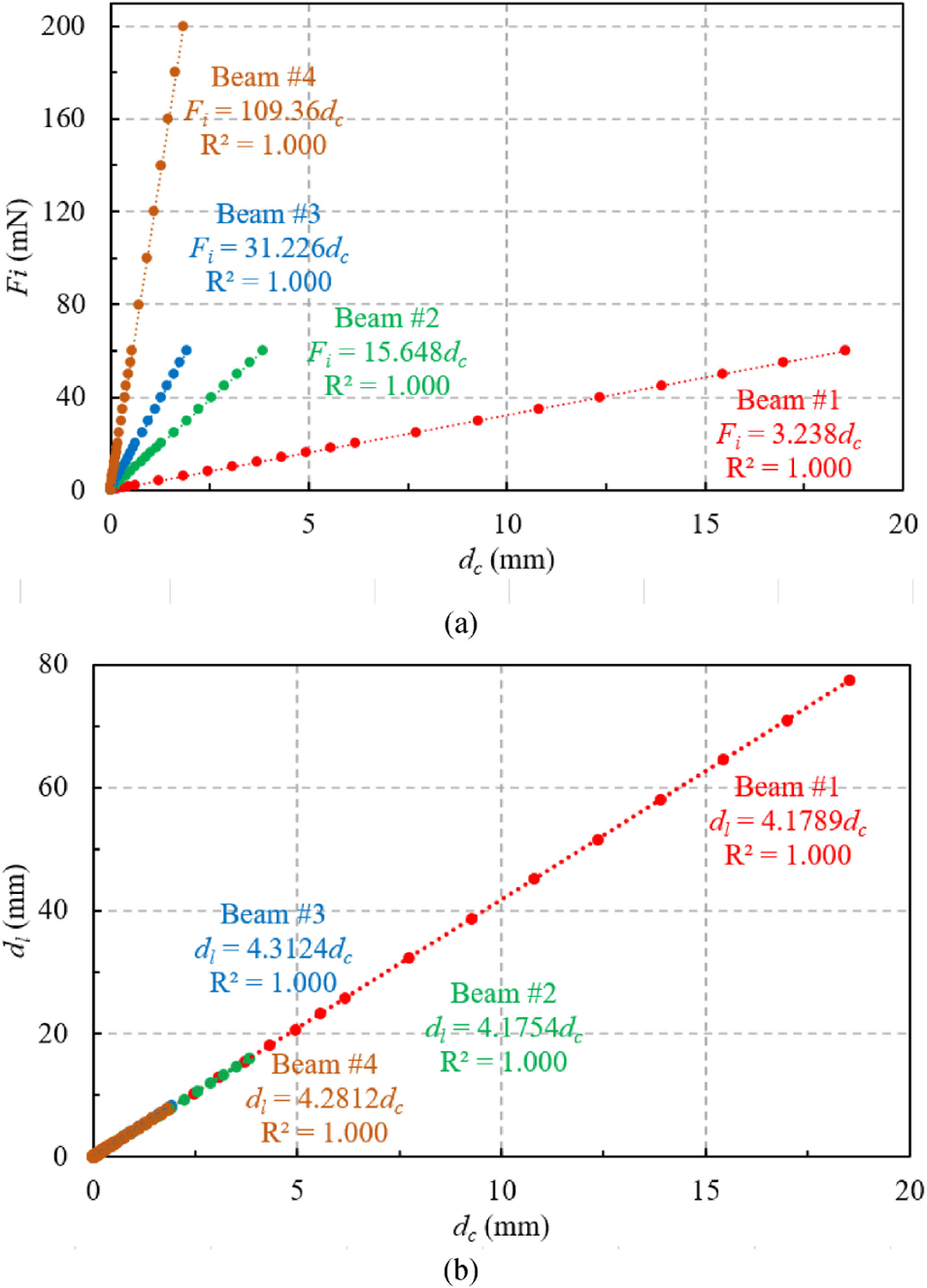
Calibration results of mapping between (a) *F*_*i*_ and *d*_*c*_ and (b) *d*_*l*_ and *d*_*c*_ for four different cantilever beam structures.

A sample deflection result of Beam #1 under 6 mN force is as shown in Fig. 2(c), which generates a correlation between the *F*_*i*_ (6 mN), *d*_*c*_ (1.853 mm), and *d*_*l*_ (7.745 mm). A summary of all these correlations for four beams under incremental loadings is shown in Fig. 3. Good linear correlations (*R*^2^ = 1.000) were achieved for all eight mappings and the correlation coefficients are summarized in Table II.

Theoretical calculation of the *F*_*i*_/*d*_*c*_ ratio (as in Table II) based on an ideal cantilever beam assumption yielded an up to 20% difference with the FEM results, indicating that gravity of the components did play an important role in the deflection due to high sensitivity of the beams and needed to be considered. This is also the reason why the vertical orientation of the cantilever beam was chosen instead of the conventional top-down insertion orientation. To validate the accuracy of the FEM, experimental calibrations were carried out by pushing the capillary tube and top plate with X-axis linear stage (to achieve specific *d*_*l*_) and recording *d*_*c*_ simultaneously. The experimentally achieved *d*_*l*_/*d*_*c*_ ratio, as listed in Table II, matched well with FEM results with discrepancies less than 2.4%, validating the FEM setup and results. Note that direct calibration of the cantilever beam system or direct validation of the FEM model by force measurement was not achievable because there was not a commercially available force sensor that could measure *μ*N-level resolution over tens and hundreds of mN-level range under a few mm displacement at *μ*N accuracy and repeatability.

Given the 0.01 *μ*m resolution and 2 mm range of the laser sensor at 171 mm height from the base used in this study, this force measurement system could measure up to 6.5 mN with 0.032 *μ*N resolution with Beam #1 and up to 218 mN with 1.09 *μ*N resolution with Beam #4.

### C. Animal Preparation

All animal experiments were carried out in accordance with the University of Michigan Institutional Animal Care and Use Committee (protocol number PRO00007803 Approved for 9/8/2017–9/8/2020 and PRO00009818 Approved for 7/13/2020–7/13/2023). For each insertion test, an anesthetized Sprague-Dawley rat was placed in a stereotaxic apparatus to fix the head and to perform a craniotomy to remove a window in the skull for microwire insertion. Anesthesia was induced and maintained with isoflurane throughout the procedure and vital signs were monitored during the entire process to make sure the animal remained within normal physiologic limits under anesthesia. Prior to surgery, carprofen was administered as an analgesic and a local anesthetic was administered to the scalp incision site prior to incision. After the animal was secured and positioned, scalp hair was removed, and the skin on the top of the head was cut by surgical scalpel to expose the skull. To prevent tissue swelling, methylprednisolone (steroid) was given intraperitoneally at 15 mg/kg of animal weight. A dental drill with a 0.5–0.9 mm diameter burr bit was then used to remove one piece of the parietal bones to create a craniotomy window to expose the membrane layers. For dura-pia penetration tests, dura mater was left undamaged after craniotomy. For pia-only penetration tests, the dura mater of the exposed hemisphere was carefully removed using fine tweezers while leaving the pia mater intact. The rat was then moved and fixed on the X-axis linear stage of the force measurement system with the craniotomy window perpendicular to the microelectrode. To maintain anesthesia, a mobile oxygen and isoflurane mixing system was used and the nose cone was kept affixed to the animal’s nose at all times. The animal and attached nosecone with both isoflurane delivery and exhaust tubing lines was transported to the top of the linear stages. The head was affixed with the craniotomy facing the cantilever beam using a custom 3D-printed head fixture with silicone padding lining to softly hold the head via pressure mount. Once the rat was affixed with gas tubing lines routed, the craniotomy was aligned with microwires to be inserted via movement of the rat with the linear stages along Y and Z directions.

During experiments, the surface of the pia or dura was cleaned with sterile 0.9% physiologic saline and re-wetted with this saline between insertions (about every 4–5 minutes) to prevent drying and to wash away any small amounts of blood to prevent clotting and consequent formation of a hardened surface outside the membrane, which would interfere with our insertion measurements. After insertion tests on one hemisphere, the exposed membrane layer was covered with triple antibiotic ointment in petroleum jelly to prevent any potential infections before conducting craniotomy surgery on the other hemisphere.

### D. Setup of the Force Measurement System

**Fig. 4.**
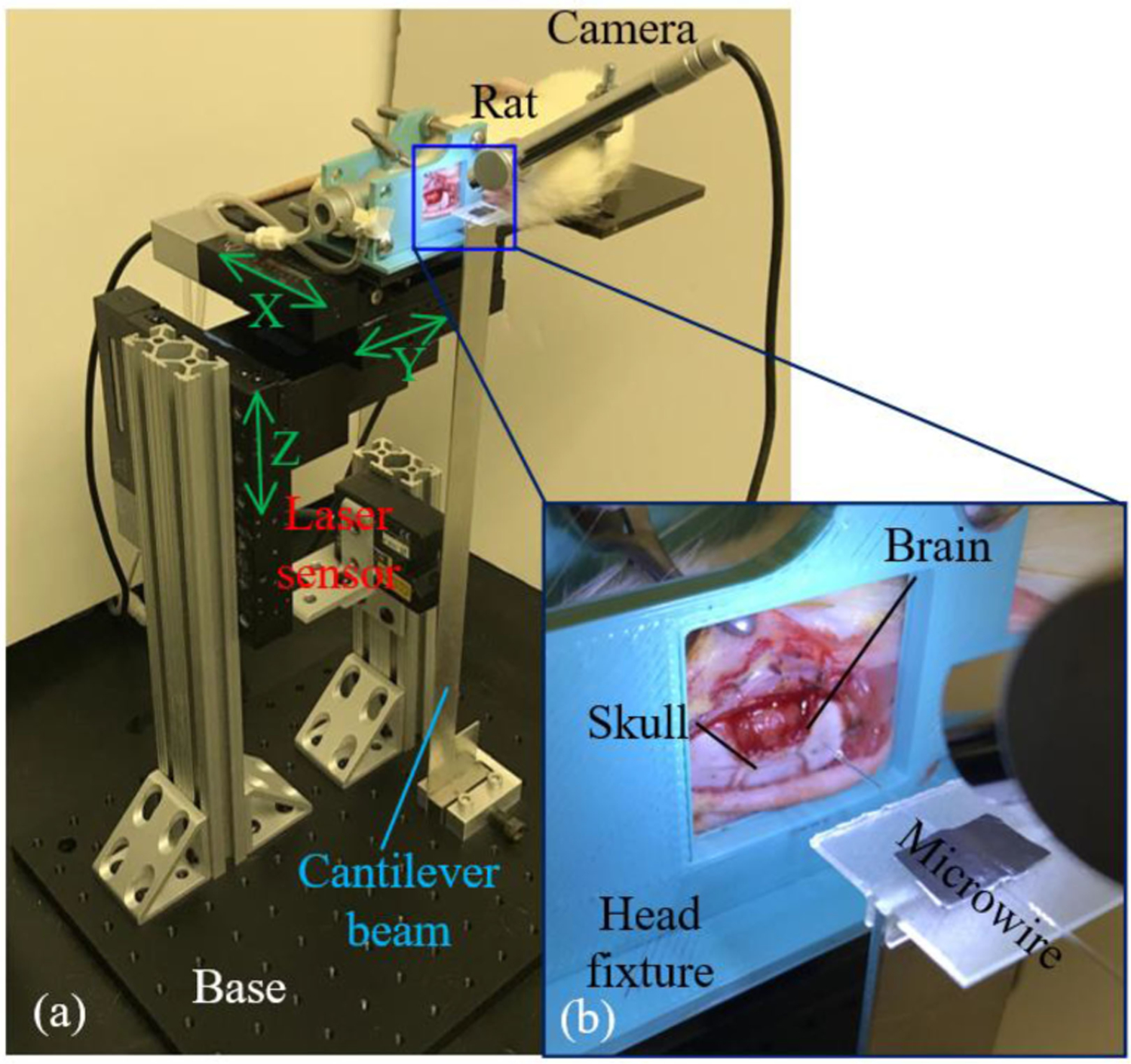
Experimental setup of microwire force measurement system: (a) anesthetized rat test setup overview and (b) close-up view of the microwire insertion into exposed rat brain.

Experimental setup of the developed force measurement system for in vivo insertion tests is shown in Fig. 4(a). The anesthetized rat was placed on its side with the head clamped by a 3D-printed fixture so that the craniotomy window was aligned with the fixture opening (as shown in Fig. 4(b)). During the experiment, motion of the entire rat was controlled by three linear stages with 25 mm travel range and 1 *μ*m resolution. By moving the rat along the X-axis, the microwire was inserted into the brain through the craniotomy window (Fig. 4(b)). Simultaneously with the laser sensor recording, dimpling of the brain tissue and microelectrode penetration into the brain could be directly observed through the digital camera.

### E. Design of Experiment

**Fig. 5.**
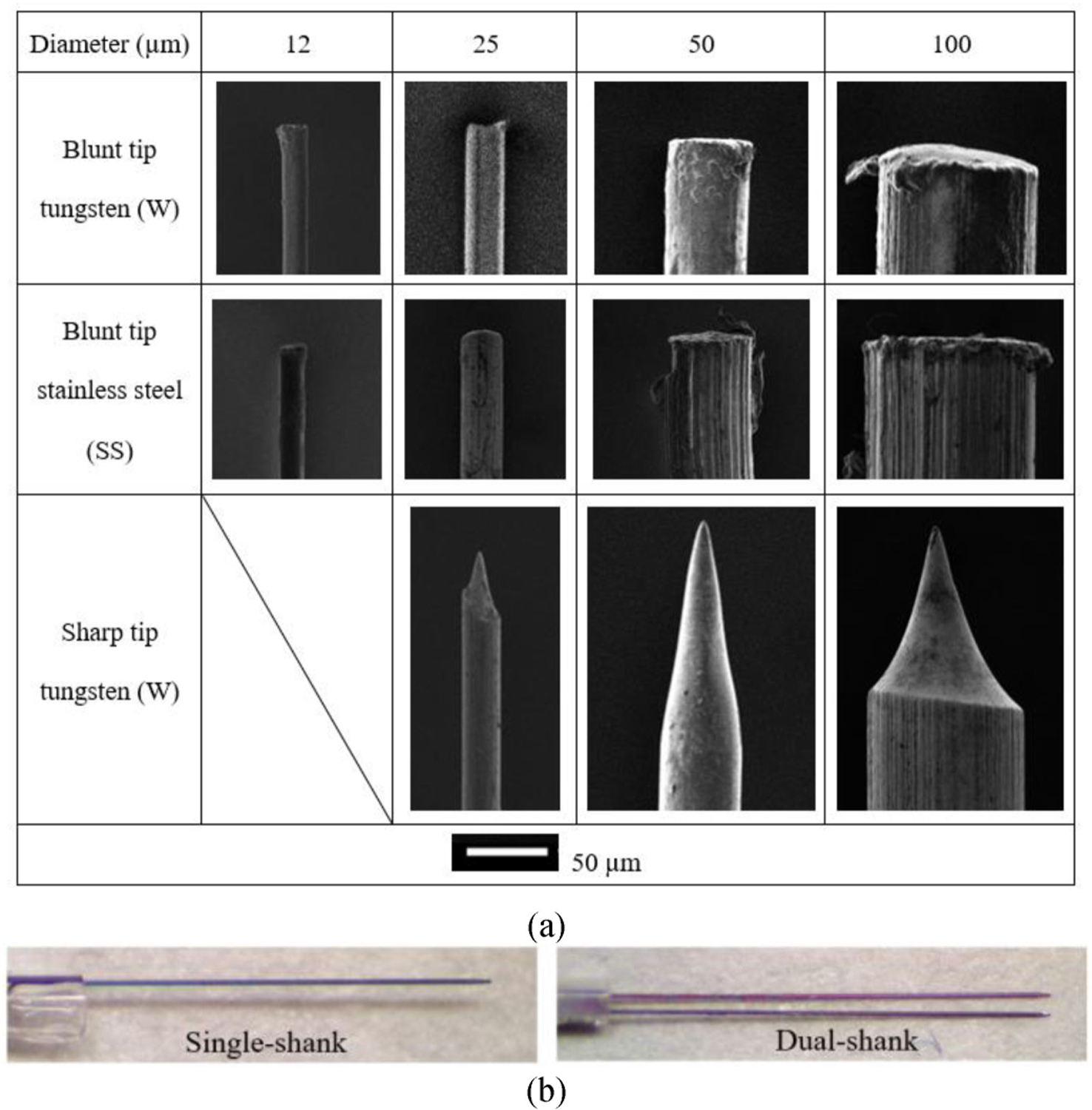
Microelectrodes used in the animal insertion tests: (a) scanning electron microscope images of various microwires and (b) single- and dual- shank silicon probes from Buzsaki64 – H64LP probe by Neuronexus fixed in capillary tube.

In this study, eight Sprague-Dawley rats (four males and four females, weighted 305–560 g) were used for in vivo insertion measurements. On each rat, one hemisphere was used for pia-only penetration tests and the other for dura-pia penetration measurements. As shown in Fig. 5(a), 11 types of microwires including two materials (AISI 304 stainless steel and tungsten), four diameters (12, 25, 50, and 100 *μ*m), and two tip geometries (polished blunt and electrochemical machining sharpened with 30° cone tip angle) were used for in vivo insertions. Overhang of microwires out of the capillary tubes were set as 1.5 mm for 12 and 25 *μ*m wires and 3.0 mm for 50 and 100 *μ*m wires. These values were chosen through preliminary trials to ensure manual microwire assembly practicability, reasonable critical buckling load for penetration, and minimal possibility of the thick capillary tube poking into the brain during cantilever beam rebounding after membrane penetration. For pia-only penetration, two new wires of each type were used for testing on each rat, yielding 22 insertion sites on the hemisphere. For dura penetration, preliminary studies showed obvious difficulties of penetration for 12 *μ*m microwires with the 1.5 mm overhang. Thus, 18 tests were conducted using the remaining nine microwire types on the dura penetration hemisphere of each rat. Insertion locations and orders were randomized on each hemisphere to minimize the variance from slight heterogeneities in brain structures and tissue property differences, though all penetrations were aimed at dorsal cortex. One exception to the random sequencing was that all the 100 *μ*m wire insertions were conducted sequentially last for each hemisphere as these tests had the highest possibilities of excessive brain trauma and bleeding, preventing other insertion trials to be conducted afterwards. Random errors were also minimized by counterbalancing factors including animal sex, left/right hemisphere, and experiment order (conducting insertions on pia-only or dura-pia penetration hemisphere first).

Major blood vessels visible from the brain surface were avoided during the insertion tests. However, challenges still existed during the insertion tests including excessive bleeding by hitting a deep vessel or pia damage during dura removal. Also, whenever significant brain edema, evident opacification, or bleeding was observed, indicating inflammation of that tissue and consequent changes in mechanical properties, experiments on that hemisphere were terminated with ongoing trial recording disregarded. As a result, 238 effective microwire insertion trials were conducted in total with 115 for pia-only penetration and 123 for dura-pia penetration.

To demonstrate the flexibility and wide application of the developed system, six pia-only penetration tests were conducted with silicon probe shanks, including two tests with single-shank, two with dual-shank (as in Fig. 5(b)), and two with a four-shank probe. The shanks were cut from an 8-shank silicon probe (Buzsaki64 – H64LP probe by Neuronexus, Ann Arbor, MI). Each shank was 50 *μ*m wide and 15 *μ*m thick with 200 *μ*m gap between adjunct shanks. The shanks overhang from the capillary tube by 3 mm, similar to conventional stereotactic-based probe insertion situations.

Preliminary insertion tests were conducted to determine the proper cantilever beam for each microelectrode device, as listed in Table III. During each insertion test, the rat brain was moved toward the microelectrode at a constant 100 *μ*m/s insertion rate (speed) until membrane rupture or microelectrode buckling occurred. This speed was suggested by Sharp *et al.* [30] as a balance between tissue stiffness and electrode adhesion. After membrane rupture or microelectrode buckling, the microwire was retracted from the brain by the X-axis linear stage.

### F. Data Collection and Processing

During each insertion, the cantilever beam deflection was monitored by the laser displacement sensor (Model LK-G10 by Keyence, Osaka, Japan). Displacement sensing data was sampled and stored at 500 Hz rate by a laser sensor controller with data storage (Model LK-G3001 by Keyence, Osaka, Japan). At the end of each insertion, the recorded displacement data set of *d*_*c*_ with 2 ms time increments were sent to a data-acquisition system for storage and further processing. Based on calibration results in Table II, the recorded *d*_*c*_ was then converted to force *F*_*i*_ and top plate displacement *d*_*l*_ at each time increment. The corresponding X-axis linear stage movement (*d*_*t*_) was calculated by multiplying the constant feed rate (100 *μ*m/s) by the insertion time. The *d*_*l*_ and *d*_*t*_ was then implemented in Equation (2) to find the displacement (*d*_*i*_), which represented the tissue dimpling before membrane rupture.

## III. Data Analysis and Results

### A. Microwire Insertion Buckling Rate

Out of the 238 effective microwire insertion tests conducted, the microwire penetrated the membrane in 215 trials and buckled in 23 trials, including 3 buckled trials for pia-only penetration and 20 for dura-pia penetration. Failure cases and buckling rates (expressing the number of buckled cases as a percentage of all trials) of each microwire type under each membrane penetration condition are presented in Table IV.

### B. Microwire Force Profile During Insertion

**Fig. 6.**
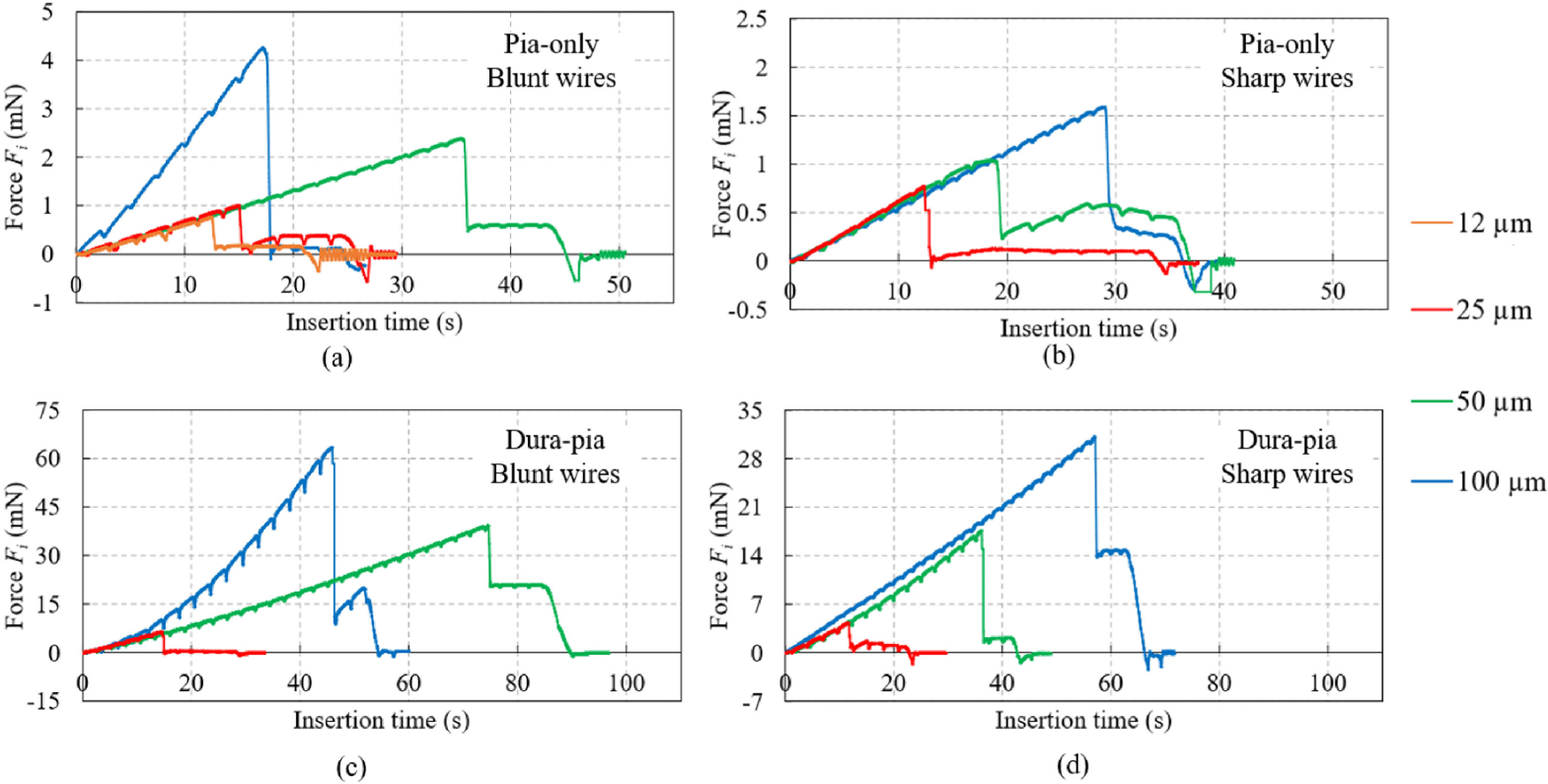
Sample force profiles of tungsten microwires: (a) blunt and (b) sharp microwires for pia-only penetration and (c) blunt and (d) sharp microwires for dura-pia penetration.

Sample profiles of the microwire force vs. insertion time in in vivo anesthetized rat are as shown in Fig. 6. Force vibrations with a periodicity of 1 – 2 seconds were observed for in vivo tests of all microwires, which showed the animal’s breathing under anesthesia [32]. For each insertion, the process start time was considered to be the moment when the microwire touched the brain membrane surface. An initial rise in the force resulted from the microwire compressing and deforming (dimpling) the membrane without penetration, which caused a linear increase in the force. Slope of this initial rise mainly depended on the beam bending stiffness as the same insertion rate (100 *μ*m/s) was used for all tests. Once the microwire ruptured the membrane, the force dropped suddenly. These force-drop points were captured to determine the membrane rupture force (insertion force) and the membrane dimpling at rupture based on (3). Unlike in previous studies [28]–[31] where the microwire stayed fixed on the rigid force sensor after penetration, in this study, the cantilever beam also bounced back after rupture together with the brain tissue rebounding. Such beam bouncing further inserted the microwire into the brain and generated a flat or slightly increasing force profile composed of the friction, cutting, and tissue clamping forces [33]. The interaction between beam bouncing, brain tissue rebounding, brain retraction, and complex brain structure (e.g., blood vessel) at the specific insertion location might cause variance (e.g., 50 *μ*m wire in Fig. 6(b) and 100 *μ*m wire in Fig. 6(c)) in this section of force profile. The force dropped again when the microwire retraction was initiated and the force might become negative because of frictional forces preventing the wire from extraction.

### C. Membrane Rupture Force and Dimpling Depth Results

**Fig. 7.**
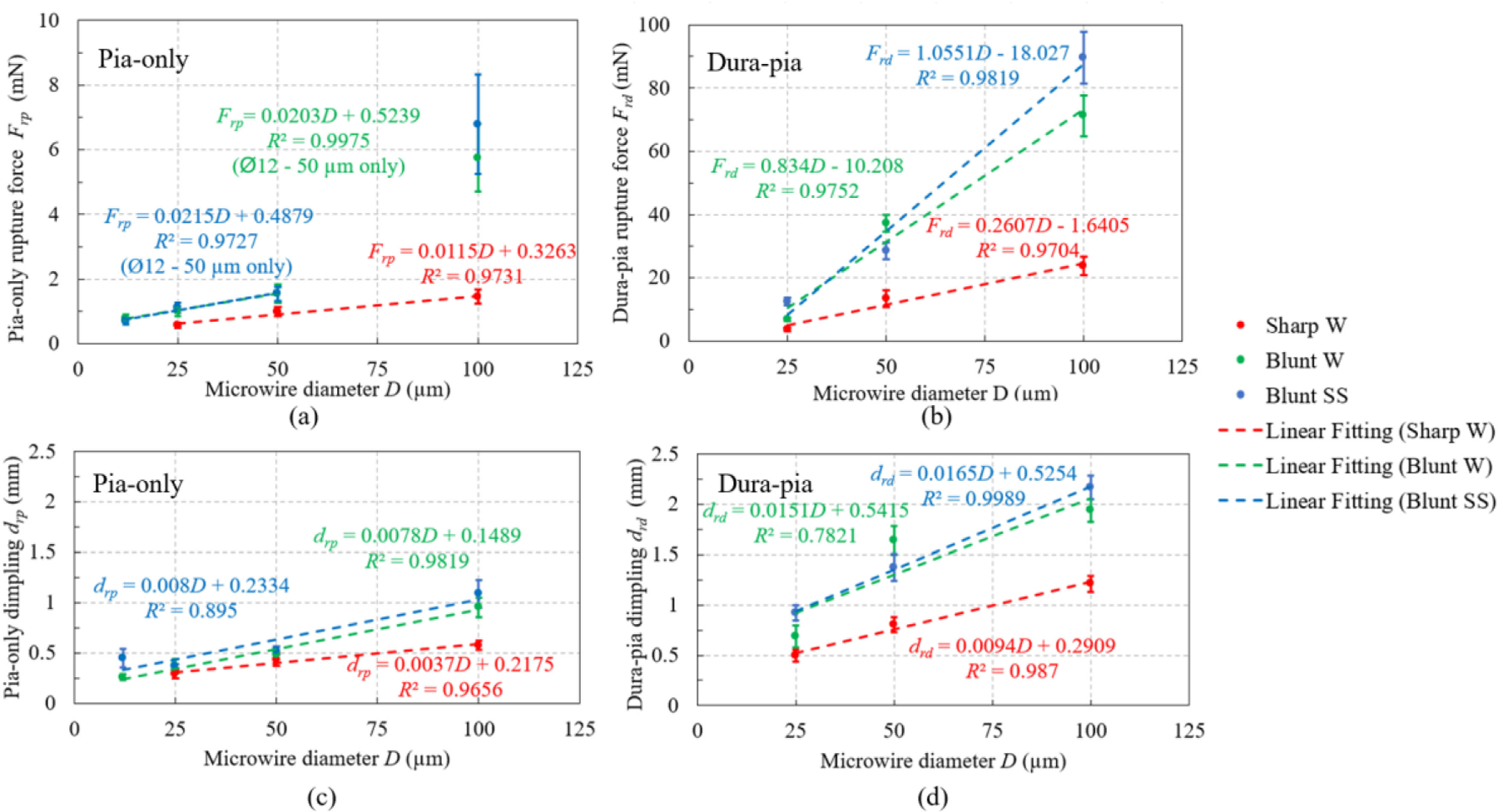
The (a) pia-only and (b) dura-pia rupture force during microwire insertions and dimpling depth at (c) pia-only and (d) dura-pia penetration by different microwires. Average value of each condition is plotted with standard error as the error bar.

Rupture force and dimpling depth at membrane rupture obtained from force profiles of all 215 successful trial are shown in Fig. 7 in terms of average value and standard error of each insertion condition. Overall speaking, dura-pia rupture force was an order of magnitude higher than pia-only rupture forces (note scale of vertical axes in Figs. 6, and 7(a), and (b)) while the dimpling depth was about 2–3 times larger. As shown by the linear curve fittings based on the average value of each dataset in Fig. 7, for both in vivo pia-only and dura-pia penetration of rat brain, both the dimpling depth and rupture force are linearly related to the microwire diameter (instead of the cross-sectional area) with *R*^2^ *>* 0.97 for rupture forces and *R*^2^ *>* 0.78 for dimpling depth. Both rupture force and dimpling depth increased with larger microwire diameter and decreased by sharpened microwires.

### D. Statistical Analysis on the Effect of Microwire Parameters

To investigate the effects of microwire parameters on the membrane rupture process, analysis of variance (ANOVA) was conducted with microwire material (tungsten or stainless steel), tip geometry (sharp or blunt), and diameter (12, 25, 50, 100 *μ*m) as three independent variables. Six ANOVA were conducted with the rupture force, dimpling depth, and dimpling-to-rupture force ratio under both pia-only and dura-pia penetration conditions as the dependent variable. The results are as summarized in Table V and a *p* value *<* 0.05 was considered statistically significant in this study, which is indicated in bold in the table. In every trial, the tip geometry and wire diameter significantly impacted rupture force, dimpling depth, and the ratio of dimpling depth to rupture force.

### E. Correlation Between Rupture Force and Dimpling Depth

Without an easily accessible force measurement system, dimpling depth has been commonly used by the neuroscience community to indirectly evaluate the force. Among the force profile, the membrane rupture force is of specific interest as it leads to potential buckling of the microelectrode. The Pearson product-moment correlation coefficient (*r*) was calculated between the rupture force and dimpling depth at rupture. Given the ANOVA result that both diameter and tip geometry significantly impact the membrane dimpling-to-rupture force ratio, seven different data sets were used to calculate the *r* of pia-only penetration for: (1) all insertions, insertions with (2) sharp or (3) blunt microwires only, and insertions with (4) 12 *μ*m, (5) 25 *μ*m, (6) 50 *μ*m, or (7) 100 *μ*m diameter microwires only. Same analysis was conducted for dura-pia penetrations except for the 12 *μ*m diameter set. The results are summarized in Table VI in terms of sample size (*n*), *p*-value (*p*), and Pearson correlation coefficient (*r*).

Statistically significant strong correlations (*p <*0.05 and absolute value of correlation coefficient *>*0.7), as indicated in bold, were only observed for pia-only penetrations under same tip geometry or with 100 *μ*m diameter. The correlation decreased with smaller diameter for pia-only penetration and only moderate correlation (absolute value of *r* between 0.3 and 0.7) existed for most dura-pia penetrations.

### F. Silicon-Based Probe Shank Insertion Results

**Fig. 8.**
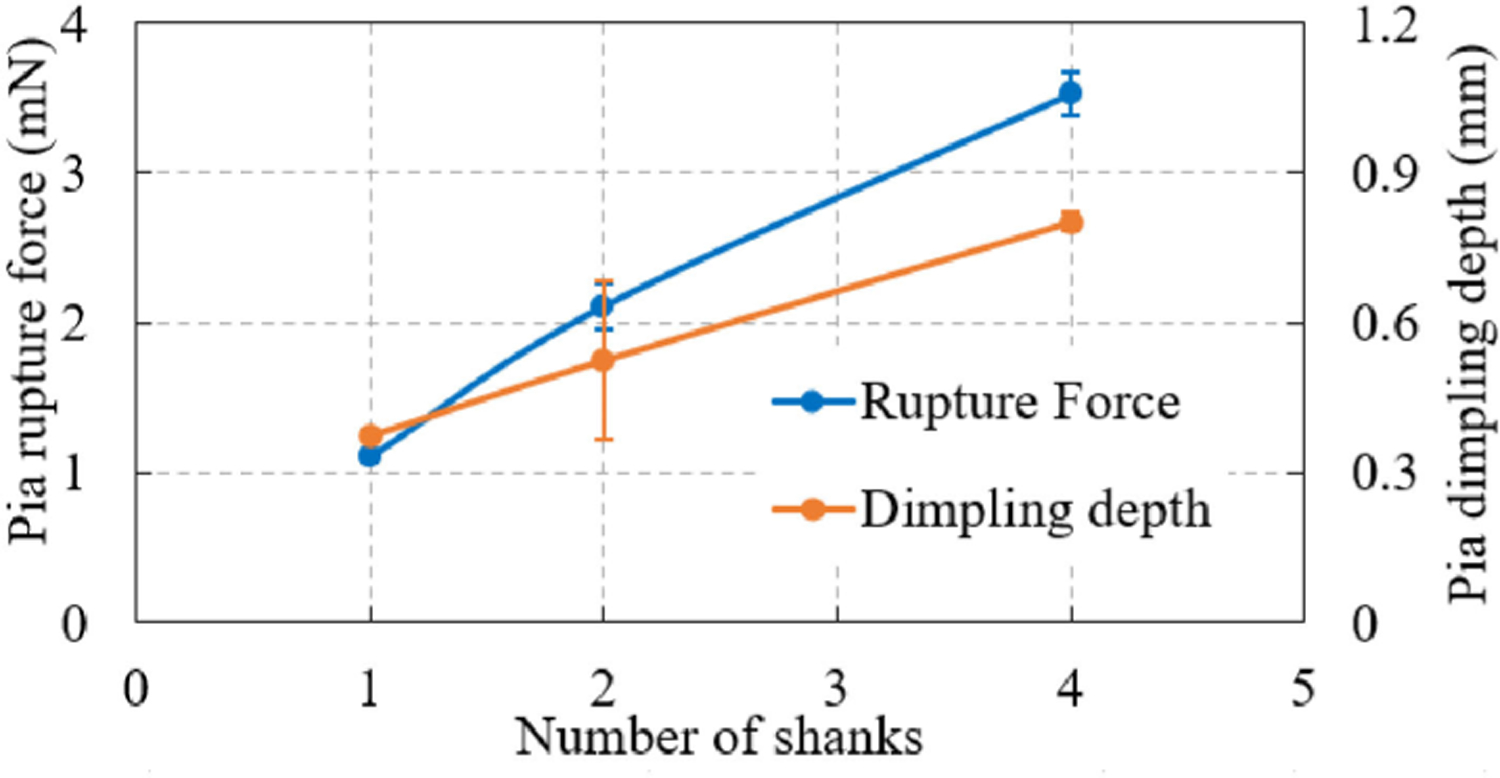
Rupture force and dimpling depth of pia-only penetration by silicon probes containing various number of shanks. Average value of each condition is plotted with standard error as the error bar.

Rupture force and dimpling depth at pia-only penetration by silicon probe shanks are as shown in Fig. 8. All six trials had pia penetration without buckling. It can be seen that due to interactions between shanks, rupture force and dimpling depth caused by dual- or four-shank devices tended to be smaller than twice or four times of the values generated by single-shank devices. It is worth noting that these experiments were conducted to demonstrate the wide application of the developed force measurement system. The six tests with sample size of two for each case may not be enough to draw any statistically significant conclusions in terms of absolute rupture force and dimpling depth values.

## IV. Discussion

### A. Flexible Configuration for Experimental Quantification of In Vivo Forces

First, we demonstrated successful use our novel *μ*N-resolution system to quantify in vivo force profile through either dura mater or pia mater in anesthetized rats. Such a system could be integrated during the surgical implantation of microelectrodes to measure the force profile and evaluate the buckling possibility during pia/dura penetration of specific microelectrode design. While our system does not have the nano-newton sensitivity of another recent system [31], but has more than sufficient force resolution range (*μ*N to as small as 32 nN) for these insertion measurements at sub-mN scale.

Key advantages of the developed force and dimpling depth measurement system is the easily accessible components and the flexibility in configuration.

Major components of the system are either commercially available (motorized linear stage, laser displacement sensor, capillary tube, metal sheets for cantilever beams, T-slot aluminum extrusions, and corresponding brackets and fasteners) or could be fabricated easily in a lab environment (3D-printed top plates and rat head fixtures). A custom designed and 3D-printed animal support fixture that could be tilted would even allow force evaluation under different orientations, if needed. Such design makes the system easily duplicable and adaptable by the neuroscience community.

It is also adaptable to varying force range and resolution needs. This study showed that by using cantilever beams of different sizes and materials, large force measurement range could be covered to evaluate the pia or even dura penetration forces of both microwires and silicon-based multi-shank microelectrode arrays. For custom force measurement needs (e.g., for single 7 *μ*m diameter sharpened carbon fiber or bulk silicon devices like the Utah Array), the user could perform a few easy modifications to achieve a larger range or higher system resolution: (1) choice of a laser sensor with higher range and resolution for higher force measurement range and resolution, (2) usage of a different cantilever beam material/geometry (more flexible beam for higher resolution and stiffer beam for higher range), and (3) change the position of the laser sensor along the cantilever beam height direction (moving up for higher resolution and moving downward for larger range).

During the force measurement by this cantilever beam-based system, deflection of the beam could slightly tilt the microelectrode and lead to a measurement error. In this specific study, trials with the largest rupture force (thus largest deflection and tilting angle) using each beam was analyzed and the calculated maximum tilting angle and resulting measurement error are as listed in Table VII. The largest rupture force recorded during animal trials using each beam was applied at the capillary tip in the corresponding beam FEM. The max tilting angle (*α*), as defined in Fig. 1(b), was extracted from the simulation results by measuring the angle between the horizontal plane and the top plate under cantilever beam deformation. The measurement error caused by the tilting angle *α* was then calculated as:

(4) Measurement error=1−cosα

It can be seen from Table VII that, by selecting a beam of proper stiffness and length, the tilting-induced force and dimpling depth measurement error was shown to be negligible (less than 0.05%) with maximum tilting angle at rupture less than 2°. It is worth mentioning that the beam tilt could also lead to microwire orientation change from the original insertion direction. But since the major tilting happened before the membrane rupture, it was compensated by either slight bending of the microwire or tip sliding on the membrane surface. Brain trauma caused by tilted insertion (of less than 2°) after brain rupture was negligible.

### B. Effect of Microwire Design on Membrane Penetration

ANOVA results showed that both microwire diameter and tip geometry have statistically significant impacts on membrane rupture force and dimpling depth. The linear relationship between microwire diameter and membrane rupture force/dimpling depth found in this study aligned with the ones observed by Obaid *et al.* [31] on pia penetration of ex-vivo mouse brain but we extended it, for the first time, to in vivo rat brain insertion and also for dura penetration. The linear correlation with the diameter instead of the cross-sectional area may be due to the fracture mechanism of the anisotropic fiber structure in the membrane layers. Rupture forces of 100 *μ*m diameter blunt microwires in pia-only penetrations were higher than the overall linear trend in this study, which was likely due to the experiment design that 100 *μ*m diameter insertion trials were set to be conducted at the end of the experiments, leading to possible changes in pia material properties over time. The large rupture size needed for 100 *μ*m blunt wires (compared to smaller diameter or sharpened wires) on that pia surface further amplified the error and led to a higher rupture force.

We quantified that sharpening of microwires could reduce the rupture force by over 40% compared to the same microwire with blunt tip. Also, sharp microwires led to smaller dimpling depth, which would be beneficial especially for shallow superficial (usually neocortical) insertions, where the dimpling depth should be smaller than the targeted recording depth to avoid over-penetration and unnecessary brain damage. Such reduction in rupture force and dimpling depth was likely due to the smaller rupture size needed for the smaller sharpened tips.

Microwire material did not significantly affect the rupture force or dimpling depth, as indicated by Table V. However, the material affected the microwire’s critical buckling load *P* (as calculated by Eq. 1) through difference in elastic modulus (410 GPa for tungsten and 200 GPa for AISI 304 stainless steel). When the rupture force and critical buckling load were close at membrane penetration (e.g., 25 *μ*m blunt wire through the dura and pia maters), stiffer tungsten microwires with higher critical buckling load would yield a lower buckling rate than the stainless steel ones, as highlighted by bold in Table IV. After membrane penetration, the force may increase again due to the frictional forces generated by larger microelectrode/brain tissue interface [31]. However, the unsupported length is also decreasing at the same time during deep insertion, leading to larger critical buckling load and thus smaller buckling possibility. As a result, the membrane rupture point is of specific interest for microelectrode buckling studies.

### C. Correlation Between Dimpling Depth and Rupture force

Given the finding that both rupture force and dimpling depth at rupture are linearly correlated with the microwire diameter (Fig. 7) and the positive correlation coefficient obtained in Table VI, the dimpling depth easily accessible through camera or microscope observations could be potentially used as a qualitative indicator for the membrane rupture force trend. A larger size microelectrode of specific tip geometry led to larger dimpling depth and membrane rupture force.

Results in this study also suggested that quantitative prediction of the rupture force based on the dimpling depth itself might not be accurate. ANOVA showed that the dimpling-to-rupture force ratio of both pia-only and dura-pia penetrations were highly dependent on the microwire diameter and tip geometry. This could be explained by the fact that the dimpling-induced rebounding force, which ultimately lead to rupture, was related to not only the membrane deformation but also the membrane-microelectrode contact area geometry and stress distribution. Both the microwire size and tip sharpness determine the contact area configuration, thus affecting the dimpling-to-rupture force ratio. A simple linear correlation between the dimpling and rupture force for all microelectrode types could not be obtained. Further correlation analysis summarized in Table VI showed a strong correlation between dimpling depth and rupture force in pia-only penetration, especially for microelectrodes with the same tip geometry or large diameter microelectrodes (100 *μ*m). This finding supports conventional dimpling measurement for evaluation of large silicon probe insertions or different insertion strategies of the same microelectrode through the pia-mater.

For smaller sized microelectrodes (*<*100 *μ*m), the correlation becomes weaker as the microwire diameter decreases, indicating the pia mater rupture to be more sensitive to random error caused by pia mater microstructures and properties of the specific insertion location as the contact area decreased. Dura mater rupture seems to be more complicated than pia mater, showing only moderate correlation under all cases despite linear correlation of both dimpling and rupture force to diameters. The reason is likely related to the membrane microstructure and thickness as well as rupture mechanics under different tip geometries, which will be further investigated.

As a summary, for pia-only penetration with less than 100 *μ*m diameter microelectrodes or dura-pia penetrations, observing dimpling depth of the membrane layer alone may not be good enough for force evaluation, buckling prevention calculation, or microelectrode design optimizations. Instead, measurement of the actual force profile to evaluate the rupture force would be recommended for quantitative analysis.

## V. Conclusion

A cantilever beam-based flexible high-resolution system for evaluation of microelectrode force and membrane dimpling depth has been developed. The easily duplicable and reconfigurable system was shown feasible for in vivo evaluation of both the pia-only and dura-pia penetration process with either microwires or silicon-based probe shanks. The following conclusions could be drawn based on microwire insertion tests conducted with the developed system:

1. For both pia-only and dura-pia penetrations, the rupture force and membrane dimpling depth at rupture are linearly related to the microwire diameter.
2. Microwire sharpening and diameter reduction have statistically significant impacts on rupture force, dimpling depth, and dimpling-to-rupture force ratio.
3. Microwire material does not significantly impact the rupture force or dimpling depth but it affects the microwire buckling rate by determining the wire critical buckling load through its elastic modulus.
4. The membrane dimpling depth and rupture force are not always strongly correlated, especially for small diameter microelectrodes (*<*100 *μ*m) and dura-pia penetration cases, making the dimpling depth observation adequate for only indirect qualitative estimation of the force profile. Direct measurement of the actual force profile would be recommended for buckling prevention analysis and detailed microelectrode design optimizations.

## Figures and Tables

**TABLE I:** Design Parameters of Four Cantilever Beams Used in the Study

Beam number	Material	Length *l* (mm)	Thickness *h* (mm)	Width *b* (mm)	Flexural rigidity *El* (N∙m^2^)
1	Al-2024	375	0.5	20	0.0152
2	Al-2024	375	0.625	20	0.0297
3	Al-2024	380	0.8	40	0.1248
4	Al-6061	380	1.5	20	0.3876

**TABLE II:** Correlation Coefficients From Calibration Results

Beam		#1	#2	#3	#4
*F*_*i*_*/d*_*c*_ (mN/mm)	FEM	3.238	15.648	31.226	109.36
	Ideal cantilever	4.058	16.62	32.712	101.76
	Difference	20.21%	5.85%	4.54%	7.47%

*d*_*l*_*/d*_*c*_	FEM	4.1789	4.1754	4.3124	4.2812
	Experiment	4.2816	4.2544	4.3999	4.3775
	Discrepancy	2.40%	1.86%	1.99%	2.20%

**TABLE III:** Cantilever Beam Selection For Each Microelectrode Based on Preliminary Insertion Tests

Membrane		Pia		Pia and dura	
Silicon probe shank number	1	*#*1		-	
	2	#1			
	4	#2			
Microwire tip type		Sharp	Blunt	Sharp	Blunt

Microwire diameter (µm)	12	-	#1	-	-
	25	#1	#1	#3	#3
	50	#1	#1	#3	#3
	100	#2	#2	#3	#4

**TABLE IV:** Membrane Penetration Success Rates

		Pia-only		Dura-pia	
Microwire Type	Diameter (µm)	Penetrated */* Total trials	Buckling rate	Penetrated / Total trials	Buckling rate
Sharp W	25	10 / 11	9.1%	10 / 14	28.6%
	50	11 / 11	0	14 / 14	0
	100	10 / 10	0	14 / 14	0

Blunt W	12	8 / 9	11.1%	**-**	**-**
	25	11 / 11	0	8 / 14	**42.9%**
	50	10 / 10	0	13 / 14	7.1%
	100	11 / 11	0	12 / 12	0

Blunt SS	12	10 / 11	9.1%	**-**	**-**
	25	10 / 10	0	6 / 14	**57.1%**
	50	11 / 11	0	13 / 14	7.1%
	100	10 / 10	0	13 / 13	0

Sum		112 / 115	2.6%	103 / 123	16.3%

**TABLE V:** Analysis of Variances (ANOVA) Results of Microwire Insertions

Dependent Variable	Independent Variable	*F*	*p*
Pia rupture force	Material	0.25	0.618
	Tip geometry	12.217	**0.001**
	Diameter	24.683	**<0.001**

Pia dimpling depth	Material	1.612	0.207
	Tip geometry	8.783	**0.004**
	Diameter	39.068	**<0.001**

Pia dimpling-to-rupture force ratio	Material	0.96	0.329
	Tip geometry	4.888	**0.029**
	Diameter	3.253	**0.025**

Dura & pia rupture force	Material	0.95	0.332
	Tip geometry	40.536	**<0.001**
	Diameter	63.409	**<0.001**

Dura & pia dimpling depth	Material	<0.001	0.983
	Tip geometry	67.803	**<0.001**
	Diameter	69.422	**<0.001**

Dura & pia dimpling-to-rupture force ratio	Material	0.2	0.656
	Tip geometry	36.058	**<0.001**
	Diameter	43.315	**<0.001**

**TABLE VI:** Correlation Between Rupture Force and Dimpling Depth at Rupture

Membrane		Pia-only			Dura-pia		

Correlation result		*n*	*p*	*r*	*n*	*p*	*r*
All insertions		**112**	**<0.001**	**0.758**	103	<0.001	0.668

Tip geometry	Sharp	**31**	**<0.001**	**0.770**	38	0.069	0.298
	Blunt	**81**	**<0.001**	**0.764**	65	<0.001	0.605

Microwire Diameter	12	18	0.755	0.079	-	-	-
	25	31	0.049	0.357	24	0.001	0.615
	50	32	<0.001	0.588	40	0.048	0.314
	100	**31**	**<0.001**	**0.725**	39	<0.001	0.561

**TABLE VII:** Analysis of Error Caused By Cantilever Beam Tilting

Beam	Microwire type	Membrane	Rupture force (mN)	Max tilting angle *α*	Measurement error
1	50 µm W	Pia-only	2.96	0.92°	0.013%
2	100 µm SS	Pia-only	19.61	1.25°	0.024%
3	50 µm W	Dura-pia	55.53	1.75°	0.047%
4	100 µm SS	Dura-pia	131.99	1.22°	0.023%

## References

[1] Fried I , Single Neuron Studies of the Human Brain: Probing Cognition Cambridge, MA: The MIT Press, 2014.

[2] Mukamel R and Fried I, “Human intracranial recordings and cognitive neuroscience,” Annu. Rev. Psychol, vol. 63, no. 1, pp. 511–537, Jan. 2012. 21943170 10.1146/annurev-psych-120709-145401

[3] Henze DA , “Intracellular features predicted by extracellular recordings in the hippocampus in vivo,” J. Neurophysiol, vol. 84, no. 1, pp. 390–400, Jul. 2000. 10899213 10.1152/jn.2000.84.1.390

[4] Strumwasser F, “Long-term recording from single neurons in brain of unrestrained mammals,” Sci. (80-.), vol. 127, no. 3296, pp. 469–470, Feb. 1958. 10.1126/science.127.3296.469 13529005

[5] Schwarz DA , “Chronic, wireless recordings of large-scale brain activity in freely moving rhesus monkeys,” Nat. Methods, vol. 11, no. 6, pp. 670–676, Jun. 2014. 24776634 10.1038/nmeth.2936 PMC4161037

[6] Rose JD and Weishaar DJ, “Tapered tungsten fine-wire microelectrode for chronic single unit recording,” Brain Res. Bull, vol. 4, no. 3, pp. 435–437, May 1979. 487197 10.1016/s0361-9230(79)80022-2

[7] Williams JC, Rennaker RL, and Kipke DR, “Long-term neural recording characteristics of wire microelectrode arrays implanted in cerebral cortex,” Brain Res. Protoc, vol. 4, no. 3, pp. 303–313, Dec. 1999. 10.1016/s1385-299x(99)00034-3 10592339

[8] Nicolelis MAL , “Chronic, multisite, multielectrode recordings in macaque monkeys,” Proc. Natl. Acad. Sci, vol. 100, no. 19, pp. 11041–11046, Sep. 2003. 12960378 10.1073/pnas.1934665100 PMC196923

[9] Tseng W-T, Yen C-T, and Tsai M-L, “A bundled microwire array for long-term chronic single-unit recording in deep brain regions of behaving rats,” J. Neurosci. Methods, vol. 201, no. 2, pp. 368–376, Oct. 2011. 21889539 10.1016/j.jneumeth.2011.08.028

[10] Prasad A , “Comprehensive characterization and failure modes of tungsten microwire arrays in chronic neural implants,” J. Neural Eng, vol. 9, no. 5, Oct. 2012, Art. no. 056015. 10.1088/1741-2560/9/5/056015 23010756

[11] Karumbaiah , “Relationship between intracortical electrode design and chronic recording function,” Biomaterials, vol. 34, no. 33, pp. 8061–8074, Nov. 2013. 23891081 10.1016/j.biomaterials.2013.07.016

[12] Liao Y-F , “A simple method for fabricating microwire tetrode with sufficient rigidity and integrity without a heat-fusing process,” J. Neurosci. Methods, vol. 195, no. 2, pp. 211–215, Feb. 2011. 21182869 10.1016/j.jneumeth.2010.12.017

[13] Prasad A , “Abiotic-biotic characterization of pt/ir microelectrode arrays in chronic implants,” Front. Neuroeng, vol. 7, pp. 1–15, 2014. 24550823 10.3389/fneng.2014.00002 PMC3912984

[14] Xie K , “512-Channel and 13-Region simultaneous recordings coupled with optogenetic manipulation in freely behaving mice,” Front. Syst. Neurosci, vol. 10, no. June, pp. 1–18, Jun. 2016. 27378865 10.3389/fnsys.2016.00048 PMC4905953

[15] et al GG, “A carbon-fiber electrode array for long-term neural recording,” J. Neural Eng, vol. 10, no. 4, Aug. 2013, Art. no. 046016. 10.1088/1741-2560/10/4/046016 PMC3875136 23860226

[16] Patel PR , “Chronic in vivo stability assessment of carbon fiber microelectrode arrays,” J. Neural Eng, vol. 13, no. 6, p. 066002, Dec. 2016. 27705958 10.1088/1741-2560/13/6/066002 PMC5118062

[17] Du ZJ , “Ultrasoft microwire neural electrodes improve chronic tissue integration,” Acta Biomater, vol. 53, pp. 46–58, Apr. 2017. 28185910 10.1016/j.actbio.2017.02.010 PMC5512864

[18] Jun JJ , “Fully integrated silicon probes for high-density recording of neural activity,” Nature, vol. 551, no. 7679, pp. 232–236, Nov. 2017. 29120427 10.1038/nature24636 PMC5955206

[19] Raducanu BC , “Time multiplexed active neural probe with 1356 parallel recording sites,” Sensors, vol. 17, no. 10, Oct. 2017, Art. no. 2388. 10.3390/s17102388 PMC5677417 29048396

[20] Rios G , “Nanofabricated neural probes for dense 3-D recordings of brain activity,” Nano Lett, vol. 16, no. 11, pp. 6857–6862, Nov. 2016. 27766885 10.1021/acs.nanolett.6b02673 PMC5108031

[21] Scholvin J , “Close-Packed silicon microelectrodes for scalable spatially oversampled neural recording,” IEEE Trans. Biomed. Eng, vol. 63, no. 1, pp. 120–130, Jan. 2016. 26699649 10.1109/TBME.2015.2406113 PMC4692190

[22] Welle EJ , “Ultra-small carbon fiber electrode recording site optimization and improved in vivo chronic recording yield,” J. Neural Eng, Mar. 2020. 10.1088/1741-2552/ab8343 PMC10771280 32209743

[23] Polikov VS, Tresco PA, and Reichert WM, “Response of brain tissue to chronically implanted neural electrodes,” J. Neurosci. Methods, vol. 148, no. 1, pp. 1–18, 2005. 16198003 10.1016/j.jneumeth.2005.08.015

[24] Marshall SP , “Effects of geometry and material on the insertion oi very small neural electrode,” in Proc. 38th Annu. Int. Conf. IEEE Eng. Med. Biol. Soc. (EMBC), 2016, vol. 2016-Octob, pp. 2784–2788. 10.1109/EMBC.2016.7591308 28268896

[25] Chen L , “Custom skull cap with precision guides for deep insertion of cellular-scale microwire into rat brain,” in Proc. ASME 14th Int. Manuf. Sci. Eng. Conf, Jun. 2019, 10.1115/MSEC2019-2967.

[26] Patel PR , “Insertion of linear 8.4 *µ*m diameter 16 channel carbon fiber electrode arrays for single unit recordings,” J. Neural Eng, vol. 12, no. 4, Aug. 2015, Art. no. 046009. 10.1088/1741-2560/12/4/046009 PMC4789140 26035638

[27] Jensen W , “Measurement of intrafascicular insertion force of a tungsten needle into peripheral nerve,” in Proc. Conf. Proc. 23rd Annu. Int. Conf. IEEE Eng. Med. Biol. Soc, 2001, vol. 3, pp. 3108–3109.

[28] Jensen W, Yoshida K, and Hofmann UG, “In-vivo implant mechanics of flexible, silicon-based ACREO microelectrode arrays in rat cerebral cortex,” IEEE Trans. Biomed. Eng, vol. 53, no. 5, pp. 934–940, May 2006. 16686416 10.1109/TBME.2006.872824

[29] Haj Hosseini N , “Comparative study on the insertion behavior of cerebral microprobes,” in Proc. 29th Annu. Int. Conf. IEEE Eng. Med. Biol. Soc, 2007, pp. 4711–4714. 10.1109/IEMBS.2007.4353391 18003057

[30] Sharp AA , “In vivo penetration mechanics and mechanical properties of mouse brain tissue at micrometer scales,” IEEE Trans. Biomed. Eng, vol. 56, no. 1, pp. 45–53, Jan. 2009. 19224718 10.1109/TBME.2008.2003261 PMC2855535

[31] Obaid AM , “Ultra-sensitive measurement of brain penetration with microscale probes for brain machine interface considerations,” bioRxiv, 2018, Art. no. 454520.

[32] Thomas J and Lerche P, Anesthesia and Analgesia for Veterinary Technicians Elsevier Health Sciences, 2016.

[33] Okamura AM, Simone C, and O’Leary MD, “Force modeling for needle insertion into soft tissue,” IEEE Trans. Biomed. Eng, vol. 51, no. 10, pp. 1707–1716, Oct. 2004. 15490818 10.1109/TBME.2004.831542

